# Astrocyte dysfunction following molybdenum-associated purine loading could initiate Parkinson’s disease with dementia

**DOI:** 10.1038/s41531-018-0045-5

**Published:** 2018-03-20

**Authors:** Christopher A. Bourke

**Affiliations:** Veterinary Diagnostic Laboratory, Orange Agricultural Institute, Orange, NSW Australia

## Abstract

Sporadic or idiopathic Parkinson’s disease is a movement disorder with a worldwide distribution, a long pre-clinical latent period and a frequent association with dementia. The combination of molybdenum deficiency and purine ingestion could explain the movement disorder, the distribution, the latent period and the dementia association. Recent studies in sheep have shown that molybdenum deficiency enables some dietary purines to accumulate in the central nervous system. This causes astrocyte dysfunction, altered neuromodulation and eventually irreversible central nervous system disease. Humans and sheep share the ability to salvage purines and this ability places humans at risk when they ingest xanthosine, inosine, adenosine and guanosine. Adenosine ingestion in molybdenum-deficient humans will lead to adenosine loading and potentially a disturbance to the A2a adenosine receptors in the nigro-striatum. This could result in Parkinson’s disease. Guanosine ingestion in molybdenum-deficient humans will lead to guanosine loading and potentially a disturbance to the guanosine receptors in the hippocampus, amygdala and ventral striatum. This could result in dementia. The molybdenum content of the average daily diet in the United States is 0.07 ppm and in the United Kingdom 0.04 ppm. Central nervous system disease occurs in sheep at <0.04 ppm. Consistent with the role proposed for molybdenum deficiency in Parkinson’s disease is the observation that affected individuals have elevated sulfur amino acid levels, depressed sulfate levels, and depressed uric acid levels. Likewise the geographical distribution of Parkinson’s dementia complex on Guam corresponds with the distribution of molybdenum-deficient soils hence molybdenum-deficient food gardens on that island.

## Introduction

**Fig. 1:**
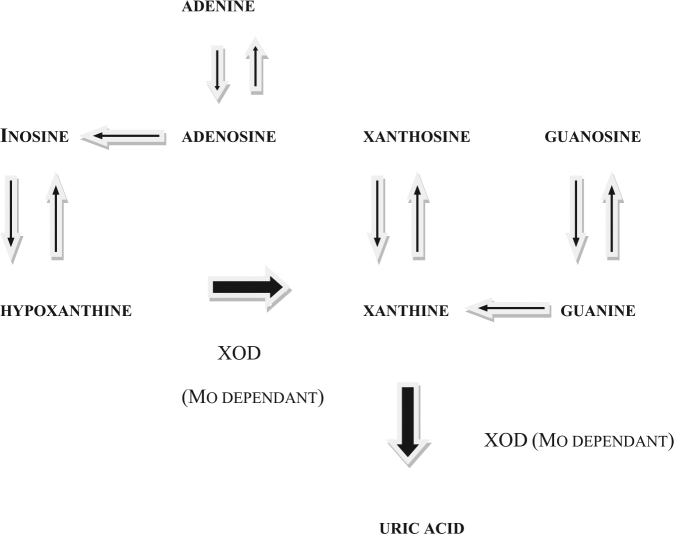
Purine catabolism in humans and sheep. XOD is the molybdenum-dependant enzyme xanthine oxidase-dehydrogenase. Molybdenum sufficiency produces a different outcome to molybdenum deficiency. *Outcome for Mo-sufficient humans and sheep:* (1) Xanthine oxidase-dehydrogenase activity is high. (2) Dietary purines are catabolised in the gut and liver and excreted. (3) They cannot be “salvaged” hence they do not reach the CNS. *Outcome for Mo-deficient humans and sheep*: (1) Xanthine oxidase-dehydrogenase activity is very low. (2) Dietary purines are not catabolised hence reach the general circulation. (3) Some are “salvaged” and reach the CNS the rest are excreted uncatabolised

Sporadic or idiopathic Parkinson’s disease (PD) is a movement disorder. Its worldwide distribution would suggest that its cause is commonly encountered. In the absence of any evidence that it is infectious or associated with a widespread environmental toxin, this would leave nutrition as a viable potential cause. PD typically occurs at 56 plus years, consistent with its having a long pre-clinical latent period. Both classical PD and Parkinson’s dementia complex (PDC) of Guam are frequently associated with dementia. To be credible, a proposed etiology needs to be able to explain the movement component, the worldwide distribution, the latent period, and the dementia component. The combination of molybdenum deficiency and purine ingestion could explain all of these. With the exception of molybdenum deficiency, clinical diseases have been described for all of the human nutritional deficiency and toxicity states and none are a match for PD. Recent studies in sheep have demonstrated for the first time that although isolated Mo deficiency is without clinical consequence, Mo deficiency concurrent with purine ingestion produces clinical disease.^1,2^ This is not unexpected because in mammalian physiology, Mo is required for the synthesis of the purine-catabolising enzyme xanthine oxidase–dehydrogenase and the sulfur amino acid catabolising enzyme sulfite oxidase. Consequently Mo deficiency would only be expected to cause clinical disease when the Mo-deficient individual was chronically ingesting either purines and or sulfur amino acids. Humans process sulfur amino acids differently to sheep but humans share with sheep, and very few other mammals, the ability to salvage purines and it is purine salvage that can lead to clinical disease. Rats and mice for example do not salvage purines and therefore do not share this disease risk. When Mo-deficient sheep, and presumably humans, ingest certain dietary purines, they have little or no ability to prevent them from entering the general circulation (see Fig. 1). This allows these dietary purines to be salvaged and then loaded into the central nervous system where they are able to survive catabolism and accumulate. This accumulation has been associated with astrocyte dysfunction and, following a very long latent period, chronically progressive irreversible CNS disease.^1,2^ Evidence has been presented elsewhere^3^ in support of the salvage of dietary sources of the purines xanthosine and inosine being involved in the etiology of human amyotrophic lateral sclerosis (motor neuron disease). The present perspective presents the opinion that the salvage of dietary sources of the purines adenosine and guanosine in Mo-deficient humans could initiate the process that leads to PD with dementia.

## Purine loading and astrocyte dysfunction

The endogenous purine nucleosides xanthosine, inosine, adenosine and guanosine, together with their bases xanthine, hypoxanthine, adenine and guanine, respectively, occur in some of the foods that humans eat. Dietary sources of these purines are normally catabolised by the Mo-dependant enzyme xanthine oxidase–dehydrogenase and then excreted (see Fig. 1). However in Mo-deficient individuals, there is insufficient enzyme activity to ensure catabolism at the level of the gastrointestinal tract and liver, hence these purines can enter the general circulation. In a minority of mammals including humans they can now be salvaged and as a consequence loaded into the CNS. Accumulation in the general salvage pathway would allow each ingested purine to be loaded despite some catabolism bias in favor of inosine and xanthine (see Fig. 1). Purine loading adversely affects astrocyte function. With both xanthosine and inosine loading in sheep, a biological impairment occurs that causes astrocyte degeneration and as a consequence an astrocyte dysfunction.^1,2^ Purine loading could also adversely affect astrocytes by adversely affecting the function of the purine receptors that are associated with them. A consequence of this would be either enhancement or inhibition of glutamate uptake by affected astrocytes, hence either reduced or increased extracellular glutamate levels. In this way, glutamatergic transmission would be either chronically reduced or chronically increased and the abnormal neuro-modulation that develops as a consequence would establish a protracted imbalance between glutamatergic excitation and GABAergic inhibition in the affected parts of the CNS. This process eventually gives rise to irreversible clinical disease.^1,2^ Although astrocyte dysfunction is the primary consequence of purine loading, neurodegeneration is likely to be a secondary consequence. This could occur as a result of some biological impairment that adversely affects astrocytes and their associated neurons, or be due to glutamate neurotoxicity, or due to the adverse effects of homocysteine and cysteine increases that can develop in affected parts of the CNS during the Mo deficiency-associated disease process.^1–3^ The level of purine loading required to achieve abnormal neuromodulation and irreversible CNS disease in the Mo-deficient sheep model was 25 mg per kg body weight per day (orally) over 18 weeks for xanthosine and 35 mg per kg body weight per day (orally) for 18 weeks for inosine. Highly specific, astrocyte-associated, chronically progressive and irreversible motor nervous neuro-modulation disorders developed in each study 18 to 31 months later. In the case of xanthosine^1^ the voluntary firing of the mid and fore-brain motor neurons (an inhibitory function) became dominant over the automatically-firing ponto-medullary motor centre (an excitatory function). The clinical outcome was asymmetric limb muscle weakness. In the case of inosine^2^ the voluntary firing of the upper motor neuron network that feeds into the ponto-medullary respiratory centre (an inhibitory function) became dominant over the automatically-firing pre-Botzinger complex (an excitatory function). The clinical outcome was diaphragm muscle weakness.

The clinical effects of either adenosine loading or guanosine loading have not yet been studied. However it is known that adenosine receptors are most concentrated in the nigro-striatum and those for guanosine in the hippocampus, the amygdala and the ventral striatum and this could indicate a role for adenosine in the initiation of PD and guanosine in the initiation of dementia. Such a proposal would not be in conflict with the pathologies already described in the literature for PD with dementia because the proposal only refers to the initiating factor not the changes that would follow such a primary event. Several autonomic nervous system dysfunctions are a common occurrence in PD. These manifestations appear to be caused by the neurodegenerative disease process that occurs in both the central and the peripheral nervous system.^4^ While they are not immediately explained by the disease initiating proposal presented here they presumably would be explained by the pathologies that eventually follow it.

## The predicted consequences of adenosine loading

Mo-deficient individuals who regularly ingest adenosine or adenine containing foods will gradually load their CNS with this purine. In a chronically progressive and irreversible process, the amount of adenosine in the CNS will rise and a dysfunction of the nigro-striatum could follow. This neuronal site in the brain is a high concentration area for A2a adenosine receptors and adenosine acts as an agonist at these receptors. The purine caffeine can reduce the risk of PD by operating as an adenosine A2a receptor antagonist^5^ consequently the agonist adenosine would be expected to have the opposite effect. A specific group of astrocytes regulates extracellular brain glutamate in the striatum via the A2a adenosine receptor mechanism.^6–8^

Many studies over recent decades have concluded that astrocytes control the uptake and release of extracellular glutamate and from these studies it has been assumed that astrocytes modulate glutamatergic neuronal excitation. Currently there is much dissent over the validity of the paradigm of astroglial transmitter release and modulation of synaptic transmission.^9^ Many now believe that vesicular release of glutamate at the synapse remains unproven. An alternative option that glutamate “release” occurs as a result of the reversal of directionality of astrocyte glutamate transporters remains acceptable. The clinical findings of Bourke^1,2^ using the sheep model, by their very nature indirectly support the notion that astrocytes modulate glutamatergic neuronal excitation in some very profound way despite our current lack of understanding as to exactly how this is achieved. By the way of contrast, other glial cells appear to be involved in the synthesis and release of neurosteroids, and have receptors for diazepam binding inhibitor to regulate this function.^10^ It is these glial cells that appear to control GABAergic neuronal inhibition. In normal individuals neuro-modulation strikes a balance between excitation and inhibition. In PD, glutamatergic neuronal excitation becomes dominant over GABA-ergic neuronal inhibition and this produces the chronic, irreversible, over-excitation of the lower motor pathways that occurs.

Adenosine loading would be expected to inhibit the uptake of glutamate by A2a receptor-associated astrocytes in the nigro-striatum, and this would allow the extracellular glutamate level to increase.^8^ Glutamate neurotoxicity could contribute to the degeneration of the nigro-striatal neurons that occurs.

## The predicted consequences of guanosine loading

Mo-deficient individuals who regularly ingest guanosine or guanine-containing foods will experience reduced excretion and excessive salvage of this purine. In a chronically progressive and irreversible process, the amount of guanosine in the CNS will increase. The areas of guanosine accumulation would be the hippocampus, the amygdala and the ventral striatum in the cerebral cortical forebrain. This is because these sites are centers of guanine deaminase activity,^11^ and guanosine receptors appear to occur here^12^ and to form a guanine-based purinergic system.^13^ Groups of astrocytes in the cerebral cortical forebrain have the ability to regulate extracellular brain glutamate and this is presumably via a guanosine receptor mechanism. Guanosine enhances glutamate uptake by astrocytes^14^ and this process would be expected to cause a reduction in extracellular glutamate levels. Guanine-based purines are known to affect cognition and memory, and have important trophic functions affecting the maintenance of neural cells.^13^ Dementia is characterized by difficulties with cognition and memory. It is possible that dementia is initiated by both guanosine loading and the astrocyte-associated glutamate disturbance that follows it, but exactly how this triggers the cascade of events that eventually give rise to the neurodegeneration associated with this disease is unclear. One possibility is that guanosine loading causes some biological impairment that adversely affects both the astrocytes and their associated neurons,

## Purine loading and astrocyte-associated PD gene mutations

Booth et al.^15^ have reviewed the role of astrocyte dysfunction in PD by looking at both the genes that are causative in the development of PD, and the aspects of astrocyte biology in which they have been implicated. They have proposed that some disruption of astrocyte biology is involved in neuron degeneration in PD. There are at least 17 monogenic mutations implicated in the development of PD and proteins encoded by eight of these play a role in astrocyte biology. Astrocyte-associated glutamate uptake and release is a critical part of the purine loading mechanism being proposed in the present perspective. Adenosine will inhibit and guanosine enhance glutamate uptake. Consequently with respect to adenosine, it is of interest that both the DJ-1 protein associated with the PARK7 gene and the alpha-synuclein protein associated with the SNCA gene have been implicated in the astrocyte function of glutamate uptake. More specifically both adversely affect astrocyte-specific glutamate transporters hence inhibit glutamate uptake.

## Mo deficiency

Because isolated Mo deficiency has not been associated with clinical disease, it has been assumed that humans eat enough Mo in their daily diet to meet all of their needs. Findings from sheep studies^1–3^ would challenge this assumption. Human Mo ingestion rates have been established in two major surveys: one in the United States^16^ and the other in the United Kingdom.^17^ It can be calculated from the results of these two surveys that the Mo content of the daily average diet in the United States is 0.07 ppm and that of the United Kingdom 0.04 ppm. However, CNS disease occurs in Mo-deficient sheep that are ingesting dietary purines, when the dietary Mo level falls below 0.04 ppm.^1–3^ On this basis it is likely that a substantial number of US and UK citizens have daily Mo ingestion rates that are too low to avoid the risk of developing CNS disease consequent to dietary purine ingestion. It would be anticipated that the higher Mo ingestion rate in the US, relative to the UK, should be associated with a lower incidence of PD in the US. Because no comparative studies of PD incidence in the US versus the UK have been published, this is difficult to resolve with certainty. However there is some evidence to suggest that the incidence in the US may in fact be lower than that in the UK. Van Den Eeden et al.^18^ have determined the incidence in Northern California to be 19.0 per 100,000 persons per year for men and 9.9 for women, with an overall incidence figure of 13.4. By comparison Duncan et al.^19^ have determined the incidence in North East England to be 17.7 per 100,000 persons per year for men and 14.4 for women, with an overall incidence figure of 15.9.

## Conclusions

Circumstantial evidence already exists for Mo deficiency being involved in the etiology of PD. Firstly, elevated plasma levels of sulfur amino acids together with depressed levels of sulfates have been reported^20^ for this disease and this is consistent with depressed activity of the Mo-dependant enzyme sulfite oxidase in these patients. Secondly Mo deficiency leads to depressed levels of uric acid (urate) and this has been a consistent finding in PD,^21^ and is indicative of depressed xanthine oxidase-dehydrogenase activity in patients with this disease. Finally, the indigenous Chamorro people on the Pacific island of Guam have historically experienced a very high incidence of PDC, and the geographical distribution of this disease on Guam matches the distribution of Mo-deficient soils on Guam. Historically the Chamorro produced most of their own food in food gardens located near their villages and the incidence of PDC was highest in the southern villages, notably Umatac and Inarajan and lowest in the western villages, notably Agana (Hagatna).^22^ The food gardens for the southern villages were located on acidic volcanic soils which are known to be very low in Mo. This can be seen in the soil test results of Miller and Sanzolone^23^ who collected 24 samples from southern Guam and determined the mean Mo content to be only 0.85 ppm (range 0.1 to 1.6). By comparison the food gardens for the western villages were located on neutral to alkaline limestone soils which are known to have moderate to high levels of Mo. This can be seen in the single comparative limestone derived soil sample that Miller and Sanzolone collected on Guam which had a Mo content of 4.3 ppm.

In most countries, including the US and the UK, a geographic bias in dietary Mo ingestion levels would not be expected, even though it occurred in the past on Guam. This is because the range of foodstuffs eaten by individuals in most countries are purchased locally but sourced from all over the country. On Guam prior to 1940 the range of foodstuffs eaten by an individual was both obtained locally and sourced locally. The diet was simple and consisted of vegetables and fruits grown in local village food gardens plus locally caught fish.^24^ From 1945 onwards, increasing numbers of foodstuffs were imported into Guam from other countries, particularly the US. The Guamanian diet gradually became more complex and eventually similar to that of the mainland US.^24^ This change would have automatically led to an increase in dietary Mo ingestion rates. In this respect, a decrease in the incidence of PDC on Guam would have been expected as the Mo ingestion rate increased and this is exactly what has happened. The annual incidence of PDC peaked for males between 1960 and 1964 at 57 per 100,000 population and for females between 1970 and 1974 at 28 per 100,000 population.^24^ It has continued to decline since and was down to 16 and 8, respectively, for males and females by 1995 to 1999. These final incidence figures are similar to those reported for PD in California in the US at the same point in time.^18^

Further studies into the potential role of Mo deficiency, ingestion of adenosine and guanosine, purine loading and astrocyte dysfunction in the etiology of PD with dementia are warranted. The Mo-deficient sheep model would be a useful tool to start this process because it could demonstrate conclusively that adenosine loading will cause motor nervous effects consistent with PD and guanosine loading dementia effects consistent with PD associated dementia. The protocol already published by Bourke for xanthosine^1^ and for inosine^2^ could be easily modified to accommodate adenosine and guanosine ingestion studies.
